# Nanobiotechnological basis of an oxygen carrier with enhanced carbonic anhydrase for CO_2_ transport and enhanced catalase and superoxide dismutase for antioxidant function

**DOI:** 10.3389/fbioe.2023.1188399

**Published:** 2023-04-14

**Authors:** Yuzhu Bian, Thomas Ming Swi Chang

**Affiliations:** Artificial Cells and Organs Research Centre, Departments of Physiology, Medicine and Biomedical Engineering, Faculty of Medicine, McGill University, Montreal, QC, Canada

**Keywords:** nanobiotherapeutic and nanobiotechnology, oxygen carriers, antioxidant, catalase, superoxide dismutase, oxygen radicals, carbonic anhydrase carbon dioxide carrier, organ preservation and transplantation

## Abstract

This is a mini review on the biotechnological aspects of the most extensively developed hemoglobin-based oxygen carriers The emphasis is on the most recent Polyhemoglobin-catalase-superoxide dismutase-carbonic anhydrase (PolyHb-CAT-SOD-CA), which is a nanobiotechnological complex that is being investigated and scaled up with the potential for clinical use as nanobiotherapeutics. Hemoglobin, a tetramer, is an excellent oxygen carrier. However, in the body it is converted into toxic dimers. Diacid or glutaraldehyde can crosslink hemoglobin into polyhemoglobin (PolyHb) and prevent its breakdown into toxic dimers. This has been developed and tested in clinical trials. A bovine polyhemoglobin has been approved for routine clinical use for surgical procedures in South Africa and Russia. Clinical trials with human PolyHb in hemorrhagic shock were effective but with a very slight increase in non-fatal myocardial ischemia. This could be due to a number of reasons. For those conditions with ischemia-reperfusion, one would need an oxygen carrier with antioxidant properties. One approach to remedy this is with prepared polyhemoglobin-catalase-superoxide dismutase (PolyHb-CAT-SOD). Another reason is an increase in intracellular pCO_2_. We therefore added an enhanced level of carbonic anhydrase to prepare a PolyHb-CAT-SOD-CA. The result is an oxygen carrier with enhanced Carbonic Anhydrase for CO_2_ transport and enhanced Catalase and Superoxide Dismutase for antioxidant functions. Detailed efficacy and safety studies have led to the industrial scale up towards clinical trial. In the meantime, oxygen carriers are being investigated around the world for use in *ex vivo* biotechnological fluid for organ preservation for transplantation, with one already approved in France.

## 1 Introduction

Hemoglobin, a tetramer, is an excellent oxygen carrier. However, in the body it is converted into toxic dimers (α1β1 and α2β2) and monomers (α1, β1, α2, β2). A number of approaches have been used to prevent this. These include intermolecular crosslinking, intramolecular crosslinking, conjugation, nanoencapsulation, and recombinant methods. These are described elsewhere (Chang et al., 2021; Chang et al., 2007). This mini review concentrates on the most extensively developed approach to nanobiotechnologic intermolecular crosslinking in the form of 1) polyhemoglobin, an oxygen carrier, 2) polyhemoglobin-catalase-superoxide dismutase (PolyHb-CAT-SOD), an oxygen carrier with enhanced antioxidant enzymes, and 3) polyhemoglobin-catalase-superoxide dismutase-carbonic anhydrase (PolyHb-CAT-SOD-CA), an oxygen carrier with enhanced antioxidant enzymes and enhanced CO_2_ carrier functions.

## 2 Polyhemoglobin

Polyhemoglobin: diacid (Chang, 1964) or glutaraldehyde (Chang, 1971) can crosslink hemoglobin into polyhemoglobin (PolyHb) and prevent its breakdown into dimers. There was no initial major interest in this until the HIV-contaminated donor blood crisis in the late 1980s led to urgent development and clinical trials. A bovine polyhemoglobin (Jahr, 2021) has been approved for routine clinical use for surgical procedures in South Africa and Russia. Clinical trials with a human PolyHb in hemorrhagic shock prehospital ambulance patients (Moore et al., 2009) were effective but with a very slight increase in non-fatal myocardial ischemia. This could be due to a number of reasons to be discussed later. PolyHb is only an oxygen carrier and could be used in conditions needing only an oxygen carrier in well-controlled surgical patients (Gould et al., 2002; Jahr, 2021). On the other hand, prehospital ambulance hemorrhagic shock patients could have different degrees of severity and may need more than just an oxygen carrier.

## 3 Oxygen carrier with antioxidant enzymes

PolyHb could act as an oxygen carrier but fails to deal with the problem of oxidative stress, including ischemia-reperfusion that produces damaging oxygen radicals (D’Agnillo & Chang, 1998). Hypoxanthine accumulates during the ischemia process. When perfused with oxygen again, xanthine oxidase converts hypoxanthine into superoxide resulting in the formation of oxygen radicals that can cause tissue injury. The antioxidant enzymes SOD converts superoxide into hydrogen peroxide which is then converted to H_2_O and O_2_ by CAT. However, in severe ischemia, even the enzymes’ activities contained inside the RBCs are not enough to prevent the tissue from ischemia-reperfusion injuries. For conditions with ischemia-reperfusion one would need an oxygen carrier with enhanced levels of antioxidant properties. One approach is to use prepared polyhemoglobin-catalase-superoxide dismutase (PolyHb-CAT-SOD) (D’Agnillo & Chang, 1998). The results on ischemic reperfusion rat models showed that PolyHb-SOD-CAT could protect the brain against the damage induced by ischemic-reperfusion in hemorrhagic shock with brain ischemia (Powanda & Chang, 2002). This result and the repeated warning (Alayash, 2005) of the need for antioxidants in oxygen carriers have led many groups to work on oxygen carriers with antioxidant properties (for example, Ma & Hsia, 2013).

## 4 Oxygen carriers with enhanced carbonic anhydrase for CO_2_ transport and catalase and superoxide dismutase as antioxidants

### 4.1 General

Clinical trials with human PolyHb in hemorrhagic shock (Moore et al., 2009) were effective but with a very slight increase in non-fatal myocardial ischemia. This could be due to a number of reasons, one of which is an increased intracelluar pCO_2_ (Sims et al., 2001; Tronstad et al., 2010). We therefore added an enhanced level of carbonic anhydrase (CA) to prepare a PolyHb-CAT-SOD-CA. The result is an oxygen carrier with enhanced Carbonic Anhydrase for CO_2_ transport and enhanced Catalase and Superoxide Dismutase for antioxidant functions. Our detailed efficacy and safety studies (Chang, 2015; Chang, 2019) have led to the industrial scale up towards clinical trial.

### 4.2 Details

**FIGURE 1:**
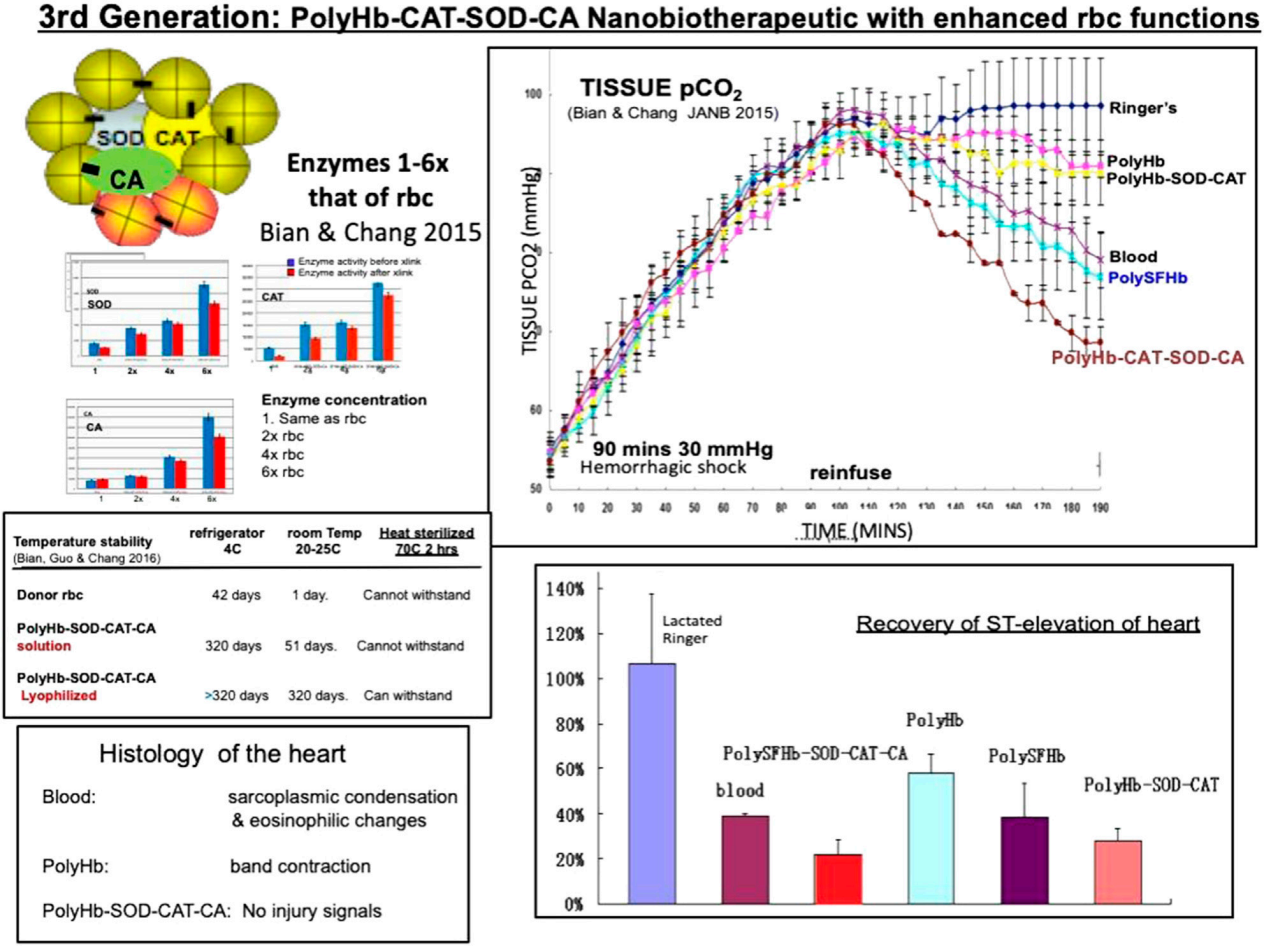
Left: PolyHb-CAT-SOD-CA with up to 6x enzyme enhancement and enzyme stability. Upper right: In a 90 min hemorrhagic shock animal model with 2/3 blood volume loss, it is superior to PolyHb and whole blood in the lowering of elevated intracellular pCO_2_. Lower right: recovery of ST elevation. Lower left: histology of the heart (From Chang 2022 with written copyright permission to reproduce this figure from the publisher Taylor and Frances.).

In addition to the needs for enhanced antioxidant enzymes discussed above, some clinical conditions also require increased transport from the tissues to the lungs for excretion. Examples include severe hemorrhagic shock and severe COVID treated by oxygenator (Chang, 2022). Increased levels of intracellular pCO_2_ are associated with myocardial ischemia as well as higher mortality rates in hemorrhagic shock (Tronstad, et al., 2010; Sims, et al., 2001). The enzyme carbonic anhydrase is the enzyme that converts CO_2_ into H_2_CO_3_ for ease of transport to the lung (Geers and Gros, 2000). This accounts for the transport of 75% of intracellular carbon dioxide to the lung. We therefore construct a new generation of nanobiotherapeutics by crosslinking hemoglobin with enhanced amounts of antioxidant enzymes (catalase and superoxide dismutase) and CO_2_ transport enzyme (carbonic anhydrase) into PolyHb-SOD-CAT-CA (Figure 1) (Bian & Chang, 2015).

The *in vivo* study was conducted with a severe hemorrhagic shock rat model by removing two-thirds of the whole blood and 60 min of sustained hemorrhagic shock at 30 mm Hg. This was followed by the reinfusion of polymerized stroma-free hemolysate (polySFHb), polySFHb-SOD-CAT-CA, whole blood, lactated Ringer’s solution, and polyHb. The results demonstrated that the infusion with 3 volumes of lactated Ringer’s solution only increased the blood pressure transiently and it then fell to 43.3 mmHg ± 2.8 mmHg. Blood (Hb 15 g/dL) maintained the MAP at 91.3 mmHg ± 3.6 mmHg while Poly-Hb-SOD-CAT-CA (Hb 10 g/dL) did so at 87.5 mmHg ± 5 mmHg, Poly-Hb-SOD-CAT at 86.0 mmHg ± 4.6 mmHg, PolySFHb at 85.0 mmHg ± 2.5 mmHg, and PolyHb at 82.6 mmHg ± 3.5 mmHg. There were no significant differences in the MAP between the HBOCs groups and the whole blood group, suggesting that all the HBOCs could maintain the MAP effectively. However, these results did not show the details of intracellular pCO_2_, recovery of heart ischemia, or histology of the heart.

Partial pressure of CO_2_ (pCO_2_) was measured upon applying the different resuscitation fluids. PolySFHb-SOD-CAT-CA was able to reduce tissue pCO_2_ from 98.0 mmHg ± 4.5 mmHg to 68.6 mmHg ± 3.0 mmHg. It was significantly more effective than lactated Ringer’s solution (ii, 98.0 mmHg ± 4.5 mmHg), blood (iii, 79.1 mmHg ± 4.7 mmHg), polyHb (iv, 90.1 mmHg ± 4.0 mmHg), polySFHb (v, 77.0 mmHg ± 5.0 mmHg), and polyHb-SOD-CAT (vi, 90.9 mmHg ± 1.4 mmHg) (Figure 1). ST elevation can be related to heart ischemia. The result of the recovery of ST elevation and the histology of the heart also shows that polyHb-SOD-CAT-CA is significantly better than blood and the other fluids in helping the cardiac tissues to recover from ischemia. (Figure 1) (Bian & Chang, 2015).

*In vitro* stability studies on polyHb-SOD-CAT-CA show that all three enzymes are stable even after 1 year of storage at 4°C or −80°C. Lyophilization makes the enzymes even more stable than the solution samples. After 1 year of storage, the freeze-dried Poly-Hb-SOD-CAT-CA still contain much of the original enzyme activities (Figure 1) (Bian & Chang, 2016).

The long-term safety and immunology studies of PolyHb-SOD-CAT-CA in rats (Guo & Chang, 2018) shows that the complex did not cause safety or immunological problems after 4 weekly 5% blood volume infusion followed by 30% volume exchange transfusion. Based on its efficacy and safety studies, the industrialization of PolyHb-SOD-CAT-CA is ongoing.

## 5 Discussions

### 5.1 General

We have reviewed the present status of 1) PolyHb, 2) PolyHb-SOD-CAT, and 3) PolyHb-SOD-CAT-CA. It would appear that each has its own area of use. For instance, PolyHb has been approved for clinical use in South Africa and Russia for surgical uses to avoid the use of HIV-contaminated donor blood. It is also being used for the *ex vivo* preservation of organs for transplantation. PolyHb-SOD-CAT-CA shows efficacy, stability, and safety in in vitro and *in vivo* studies. The results show a promising nanobiotherapeutic application of the PolyHb-SOD-CAT-CA. The next step includes scaling up while maintaining batch-to-batch consistent quality and functional efficacy. In the meantime, it is an effective *ex vivo* preservation and regeneration method for organs for transplantation.

### 5.2 *Ex vivo* use for organ and cell preservation

Studies and methods to solve the potential toxicity of many HBOCs are being carried out with promising results. *Ex vivo* uses are also showing promising results in avoiding large volumes of nanobiotherapeutics being introduced into the body. Early research on the *ex vivo* use of PolyHb for preservation and regeneration of isolated small intestine for transplantation (Razack et al., 1997) is being extended with promising results. Recent exciting uses of different types of HBOCs for organ preservation and regeneration for transplantation include the heart, kidney, liver, lung, pancreas, and small bowel (Van Leeuwen et al., 2019; Cao, et al., 2021). HBOCs can be used together with traditional preservation solutions showing very good compatibility. Studies show that HBOCs could extend the preservation time and decrease ischemic and hypoxic damage to isolated organs to some extent. The safety and efficacy using a marine source of oxygen carrier with antioxidant properties for liver and kidney preservation has been demonstrated in recent clinical trials, leading to approval in France (Le Meur et al., 2020). Recently, Sestan’s group demonstrated restored circulation and cellular activity hours post-mortem in isolated porcine brains using HBOCs as the resuscitation solution (Vrselja, et al., 2019). In the most recent exiting study published in Nature (Andrijevic, et al., 2022), they applied the OrganEx, an adaptation of the BrainEx extracorporeal pulsatile-perfusion system and cytoprotective perfusate containing HBOCs for porcine whole-body perfusion. Tissue integrity has been preserved and selected molecular and cellular processes have been restored across multiple vital organs after 1 h of warm ischemia. The most recent Polyhemoglobin-catalase-superoxide dismutase-carbonic anhydrase (PolyHb-CAT-SOD-CA) with enhanced enzyme activity could be even more effective in the preservation and regeneration of isolated organs.
